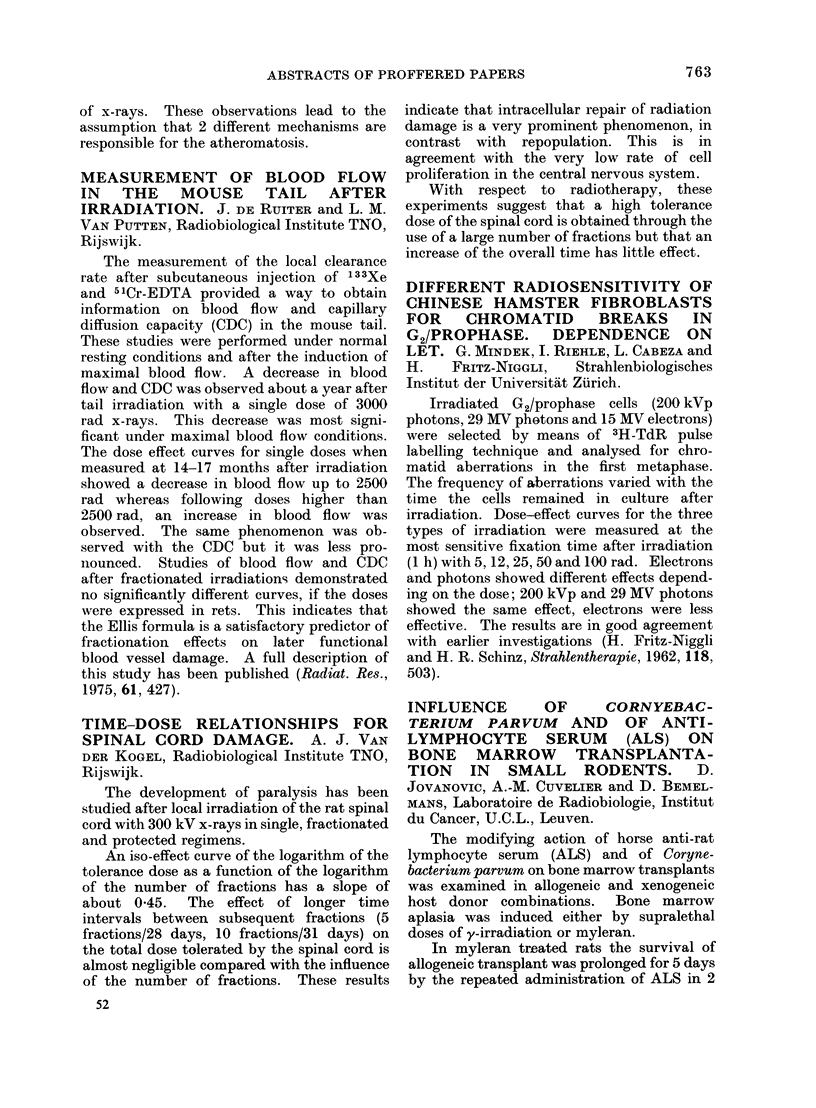# Proceedings: Different radiosensitivity of chinese hamster fibroblasts for chromatid breaks in G2/prophase. Dependence on LET.

**DOI:** 10.1038/bjc.1975.330

**Published:** 1975-12

**Authors:** G. Mindek, I. Riehle, L. Cabeza, H. Fritz-Niggli


**DIFFERENT RADIOSENSITIVITY OF CHINESE HAMSTER FIBROBLASTS FOR CHROMATID BREAKS IN $G_2$/PROPHASE. DEPENDENCE ON LET.** G. Mindek, I. Riehle, L. Cabeza and H. Fritz-Niggli, Strahlenbiologisches Institut der Universität Zürich.

Irradiated $G_2$/prophase cells (200 kVp photons, 29 MV photons and 15 MV electrons) were selected by means of $^3H$-TdR pulse labelling technique and analysed for chromatid aberrations in the first metaphase. The frequency of aberrations varied with the time the cells remained in culture after irradiation. Dose–effect curves for the three types of irradiation were measured at the most sensitive fixation time after irradiation (1 h) with 5, 12, 25, 50 and 100 rad. Electrons and photons showed different effects depending on the dose; 200 kVp and 29 MV photons showed the same effect, electrons were less effective. The results are in good agreement with earlier investigations (H. Fritz-Niggli and H. R. Schinz, *Strahlentherapie*, 1962, **118**, 503).